# Ecotourism in Traditional Cultural Landscapes: A Regenerative Lens on Stakeholder Perspectives in Vama Buzăului, Romania

**DOI:** 10.1007/s00267-026-02498-x

**Published:** 2026-05-25

**Authors:** Sandeep Joshi, Alexandra Bucur, Andrei Atomulese, Stefan Zerbe, Martin Sauerwein, Sabine Panzer-Krause

**Affiliations:** 1 University of Hildesheim, Institute of Geography, Hildesheim, Germany; 2 Babeș-Bolyai University, Faculty of Geography, Cluj-Napoca, Romania; 3 University of Applied Science and Arts, Göttingen, Germany

**Keywords:** Traditional Multifunctional Cultural Landscapes (TMCLs), Ecotourism, Regenerative tourism, Stakeholder perceptions, Eco-certified destination

## Abstract

Traditional Multifunctional Cultural Landscapes (TMCLs) are coupled socio-ecological systems in which cultural heritage, land-use practices, and ecological functions co-exist and are co-produced. Ecotourism has increasingly been promoted as a tool for sustainable development in TMCLs, yet little empirical evidence exists on how local stakeholders perceive its impacts. This study explores stakeholder perspectives on ecotourism development in Vama Buzăului, Romania an eco-certified destination, and explores its implications through regenerative tourism lens. Semi-structured interviews with twelve diverse stakeholders provided insights into socio-cultural, economic, environmental, youth outmigration and governance-related impacts. The findings reveal that stakeholders associated ecotourism with socio-cultural revitalization (community pride, festivals, gastrolocal identity) but also raised concerns regarding crowding and cultural commodification. Economic perceptions emphasized new livelihood opportunities, indirect value-chain benefits for non-tourism actors, and improved infrastructure, alongside concerns about unequal benefit distribution and rising land prices. Environmental perceptions were ambivalent: stakeholders noted improved waste management and conservation initiatives (e.g., the bison reserve) while also highlighting litter, traffic congestion, and reduced communal grazing areas. Respondents further linked ecotourism to youth retention and land re-use, though outsider investment was perceived as a potential pressure on traditional land-use patterns. Finally, local government was viewed as a key enabler through administrative support and small-scale infrastructure development, while direct financial support was considered insufficient and future needs included diversified attractions, micro-loans, and capacity building. Interpreted through a regenerative lens, these outcomes demonstrate how ecotourism in TMCLs can move beyond sustaining existing systems to actively enhancing cultural vitality, community resilience, and ecosystem health. The study highlights the pivotal role of participatory governance and local government in enabling regenerative pathways, emphasizing inclusive planning, equitable benefit-sharing, and long-term policy support. Overall, Vama Buzăului provides an illustrative case of how TMCLs can serve as real life laboratories for regenerative rural development, offering lessons for destinations seeking to align ecotourism with holistic development while avoiding unidimensional growth-driven, unsustainable ecotourism models.

## Introduction

Traditional cultural landscapes are dynamic socio-ecological systems that often bear a high degree of cultural and natural heritage (Aplin, 2007) and thus represent the confluence of tradition and nature, encompassing both tangible and intangible heritage (Pătru-Stupariu, et al., 2019). These cultural landscapes often exhibit multifunctionality in its environmental, social and economic dimensions and are referred to as Traditional Multifunctional Cultural Landscapes (TMCLs) (Zerbe, 2022). ‘Tradition’ in this context, encompasses elements such as land-use practices, architectural styles, governance structures, customs that collectively uphold cultural values being passed from generation to generation (Zerbe, 2022). Multifunctionality in this context does not merely mean that “more than one activity occurs” in a landscape. Rather, multifunctionality as an analytical concept implies the intentional or demonstrable co-existence and co-production of multiple bundles of functions at the landscape scale, instead of the dominance of a single primary function that suppresses or externalizes others (Zerbe, 2024). In the specific context of TMCLs, “multifunctional” denotes the simultaneous delivery of ecological, social/cultural, and economic functions/services rooted in long-standing land-use practices and governance/meaning systems. This is often best captured through a landscape-services framing, which explicitly integrates biophysical functions with cultural values and institutions and allows assessing synergies and trade-offs within one place-based system (Termorshuizen & Opdam, 2009; Vallés-Planells, et al., 2014). Not all landscapes are meaningfully multifunctional; in TMCLs, multifunctionality is maintained through long-standing stewardship practices and institutions that can unravel when a single use becomes dominant or when local capabilities decline. These landscapes, shaped over centuries, millennia, are not static instead they continually evolve, serving as repositories of collective memories, identities, and social cohesion (Zerbe, 2025). These collective memories include historical events, traditional practices, and cultural rituals. The identities they preserve are those of the communities that have inhabited and shaped these landscapes over generations. The social cohesion they foster is evident in the shared values, norms, and customs that bind these communities together. TMCLs provide critical insights into holistic development illustrating how cultural continuity can persist despite ongoing environmental and societal changes (Joshi, et al., 2023). They reflect the intricate balance between human activity and ecological stewardship and provide valuable models of holistic development.

Tourism has a reciprocal and complex interrelationship with the environment, relying heavily on natural and cultural resources. Hereby, ecotourism is becoming of increasing interest worldwide to cope with environmental challenges and socio-economic transformations, especially in rural areas (Buckley, 2012; Joshi, et al., 2025). Ecotourism emerged as a segment of alternative tourism development during the 1980s, driven by the perception that modern mass tourism practices were deleterious to host destinations (Koens, et al., 2009; Wondirad, et al., 2020; Mondino & Beery, 2019). The Global Ecotourism Network defines ecotourism as responsible travel to natural areas that conserves the environment, sustains local well-being, and fosters knowledge and understanding through education (Global Ecotourism Network, 2016). Ecotourism, a niche within tourism, has core objectives of preserving natural and cultural capital, supporting local rural development and promoting knowledge dissemination (Fang, 2020).The emergence of ecotourism marked a shift towards more sustainable tourism practices. As a multifaceted strategy, ecotourism prioritizes sustainable enjoyment of natural and cultural landscapes, potentially contributing to biodiversity conservation and restoration as well as community involvement in planning and cultural exchange (Mckercher, 2010; Walter, 2013; Jamaliah & Powell, 2018) (Mckercher, 2010; Walter, 2013; Jamaliah & Powell, 2018).

Although often conflated with “sustainable”, “responsible”, “nature”, “green” or “low-impact” tourism, it is crucial to conceptualize ecotourism as a distinct category within the broader tourism spectrum (Honey, 2008). In practice, this distinction is frequently operationalized through credible certification and standards that codify environmental, socio-cultural, and economic requirements and thereby help reduce “greenwashing” and ambiguity (Buckley, 2012; Font & McCabe, 2017). In the present case, ecotourism is not used only as an abstract concept but is also institutionally defined; Vama Buzăului is an eco-certified ecotourism destination within Romania’s national Eco-Romania framework administered by the Association of Ecotourism in Romania (AER, 2023) (further site details are in Section 2.1).

Beyond sustainability-oriented framings, regenerative tourism has emerged as a perspective that emphasizes place-based renewal and net-positive socio-ecological outcomes rather than only mitigating harms (Pollock, 2020; Bellato, et al., 2022; Smithwick, et al., 2023). Regenerative tourism is increasingly discussed as a paradigm that moves beyond impact-mitigation logics by positioning tourism as a place-based, living-system endeavor that seeks to enhance the regenerative capacity of coupled human–nature systems and generate net-positive outcomes over time. However, regenerative tourism does not yet have a single universal definition; rather, recent syntheses show that it is best understood through a set of conceptual pillars that recur across the literature, most notably community-centrism, an ecological worldview and living-systems thinking, meaningful multi-stakeholder collaboration, and an explicit orientation toward net positive effects (Iddawala & Lee, 2025). In this article, we therefore employ regenerative tourism not as a second descriptive framework parallel to sustainability, but as an interpretive lens that guides how stakeholder-reported changes are understood. While results are presented using the familiar socio-cultural, economic and environmental categories for transparency and comparability it should be noted that regenerative tourism is not a standalone concept rather it builds upon the concept of sustainability (Mang & Reed, 2012). Within TMCLs, a regenerative lens is analytically useful because it foregrounds whether ecotourism is perceived to strengthen local stewardship, cultural vitality, and community capability to sustain multifunctionality over time rather than producing only incremental improvements. In this study, regenerative tourism is therefore used as an interpretive lens for stakeholder reported impacts and governance constraints without expanding into a separate conceptual treatise.

Several studies have acknowledged the potential of ecotourism to facilitate the sustainable development of rural destinations (Doan, 2010; Ressurreiç, et al., 2022; Anindhita, et al., 2024). A substantial body of research examines resident attitudes and stakeholder perceptions of tourism and ecotourism impacts in rural destinations. However, TMCL-specific evidence that foregrounds how stakeholders interpret ecotourism trade-offs, governance realities, and multifunctionality dynamics that is explicitly interpreted through a regenerative lens remains comparatively limited. For instance, a recent systematic review (Joshi, et al., 2023), of rural tourism in European TMCLs indicates that research in this domain is still at an early stage and highlights a limited, sparse focus on local stakeholder perceptions within the existing literature suggesting the need for more empirical work on local stakeholder perspectives in TMCL ecotourism contexts.

The study of Vama Buzăului, a TMCL presents a unique opportunity to fill the stated research gaps. The local stakeholders’ perspectives in such a setting are crucial for understanding the real-world impacts of ecotourism on TMCLs, particularly regarding environmental, cultural, and socio-economic outcomes. Here, stakeholders are understood as the local community and the two terms are used interchangeably. This is because ecotourism activities unfold within a shared socio-spatial setting in which community members live and work, consequently, all local residents regardless of whether they are directly engaged in ecotourism economically are implicated in its impacts and outcomes and therefore constitute stakeholders.

Additionally, by examining the role of ecotourism in mitigating land abandonment and outward migration, this study offers practical insights that could inform policy and planning in similar rural TMCLs across Europe and beyond. The research emphasizes the need for inclusive and participatory approaches in developing ecotourism strategies by highlighting the importance of local stakeholder perspectives. It underscores the potential of TMCLs to serve as models for holistic development, where cultural heritage and nature conservation are harmoniously integrated within the socio-economic fabric of local communities. The main aim of this empirical study is to address these gaps by providing an in-depth analysis of local stakeholders’ perceptions regarding ecotourism’s role in the development trajectory of the TMCL.

The specific objectives are:

- To assess the perceptions of local stakeholders concerning ecotourism’s impacts (both positive and negative) on the environmental, economic, and socio-cultural dimensions of the TMCL.
- To evaluate the role of ecotourism in addressing the issue of land abandonment and outward migration in the TMCL.
- To analyze how ecotourism benefits stakeholders not directly involved in the tourism sector.
- To analyze the local stakeholder’s perception of the local government’s role in ecotourism development and identify local perspectives of future requirements for ecotourism development.

Based on our study, we interpret the results through the lens of regenerative tourism, examining whether ecotourism in TMCLS actively revitalizes cultural, ecological and socio-ecological systems.

## Materials and methods

### Study area

**Fig. 1:**
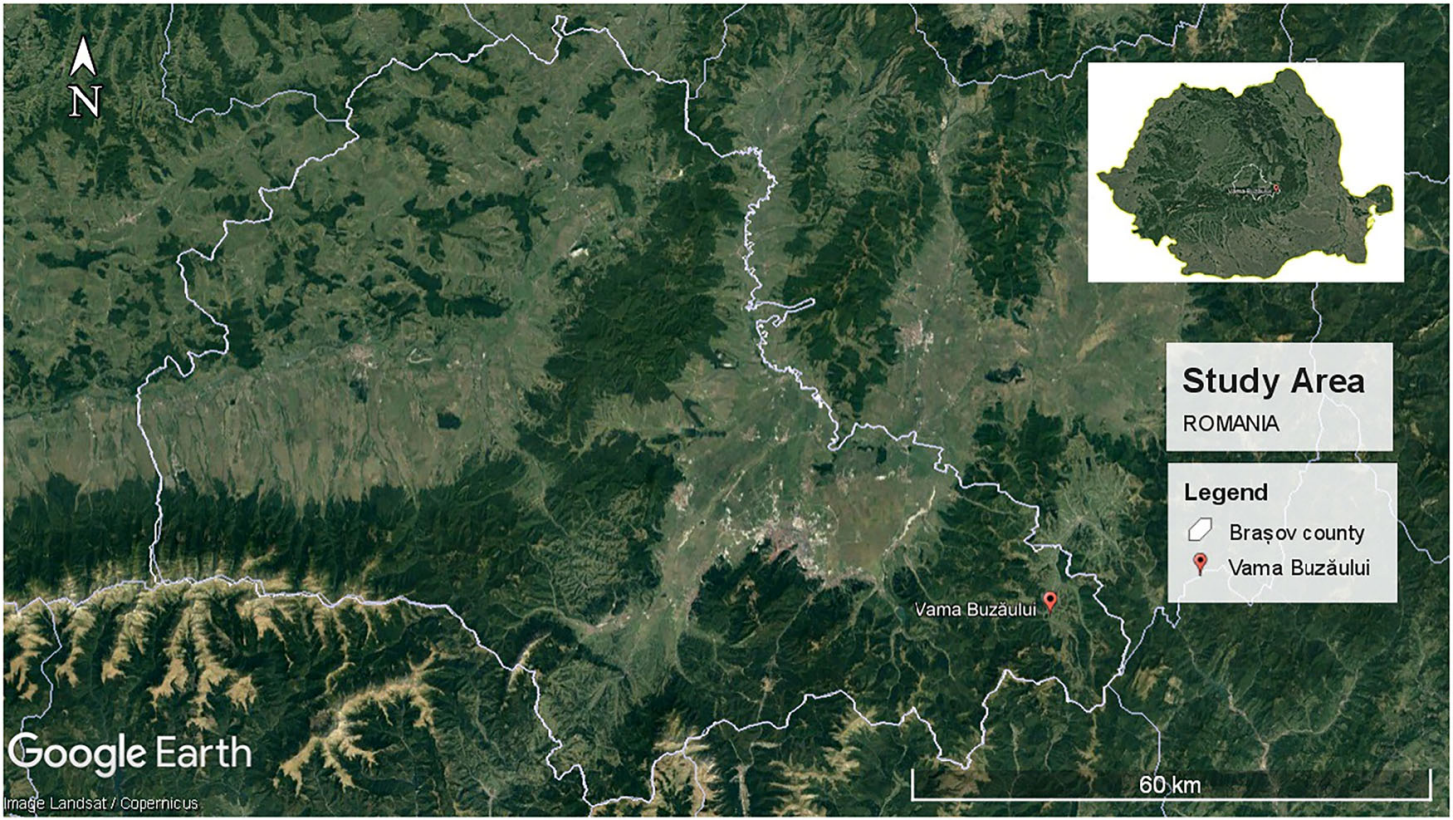
Vama Buzăului, Brasov County, Romania (Google Earth, 2025)

**Fig. 2:**
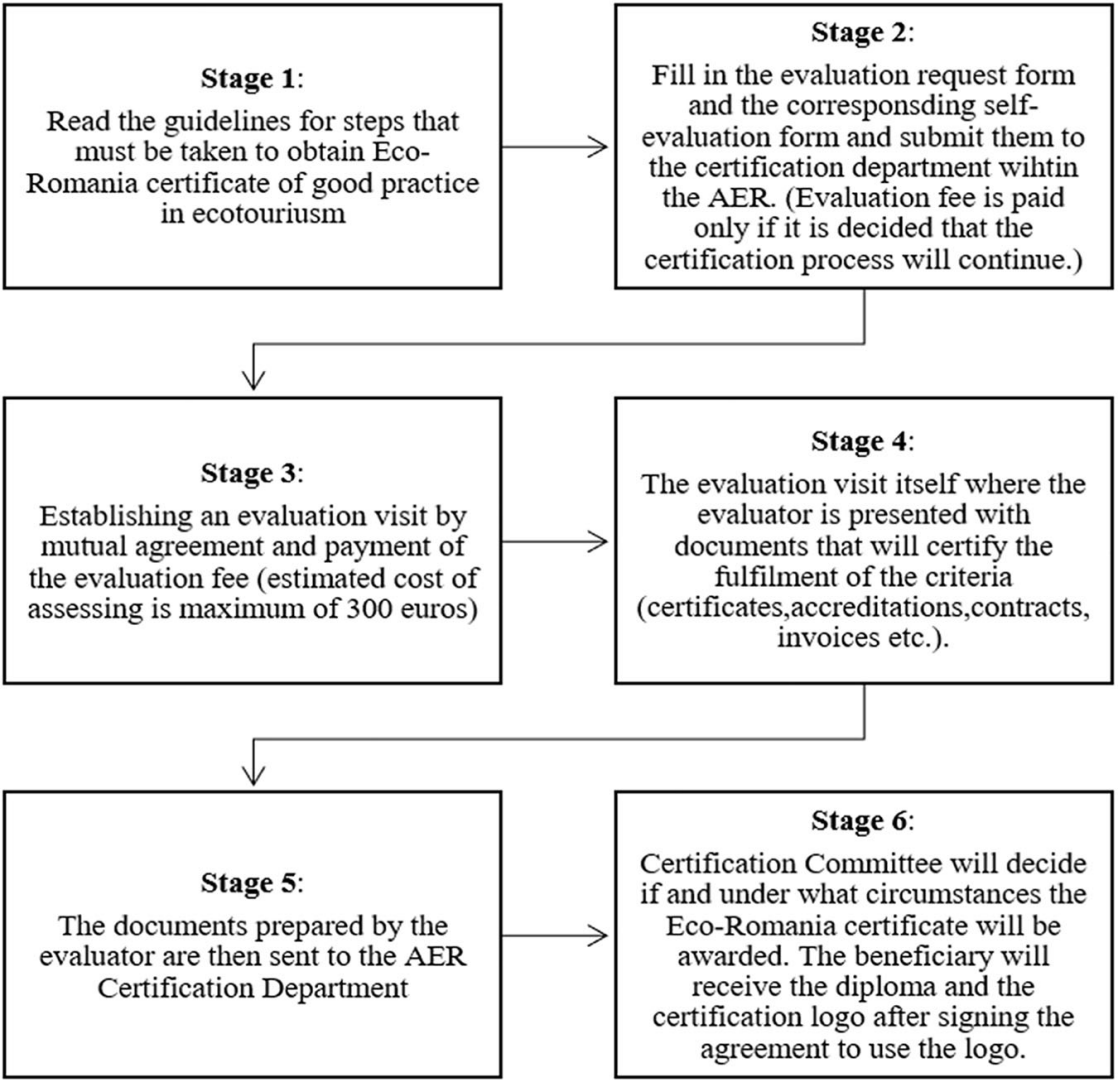
Eco-certification process of Vama Buzăului (AER, 2023)

Vama Buzăului, Brașov County in Romania Situated 45 km southeast of Brașov, Vama Buzăului is an eco-certified destination, one of only eight in Romania (AER, 2023) (AER, 2023). Figure 1 shows the location map of the study area. Figure 2 shows the eco-certification process. Vama Buzăului is characterized by a temperate continental climate with distinct seasonal variations. It experiences warm summers and cold, snowy winters, with an average annual temperature ranging between 8–10 °C. Precipitation is well-distributed throughout the year, with a slight increase during the summer months, supporting the region’s lush vegetation. The landscape is marked by a mosaic of temperate deciduous and coniferous forests (Stăncioiu, et al., 2018). Historically, Vama Buzăului served as a strategic border between the Austro-Hungarian Empire and the Kingdom of Romania, a role that has influenced its cultural identity. The destination’s name, “Vama,” meaning “customs” or “border” in Romanian, and ‘Buzau’ is the name of the river flowing through the border, symbolizing the intersection of natural and cultural elements in the area. The village’s rich history is reflected in the amalgamation of various villages and the formation of collective communes, each shaped by unique land-use practices, governance systems, and customs, which together enrich the region’s cultural heritage (VBTIC, 2023).

The community of Vama Buzăului exhibits a strong sense of social cohesion deeply rooted in its traditions and cultural practices. The village is renowned for its customary fairs, folkloric exhibitions, and performances that highlight traditional crafts and local products. Annual events like “Fiii Satului,” “Festivalul Gospodarul Vămășan,” and “Vama de la Munte” serve as vital platforms for showcasing and integrating the region’s tangible and intangible cultural heritage. Vama Buzăului is also the birthplace of Romania’s ‘Gastrolocal’ movement, where visitors can enjoy traditional dishes made from locally sourced ingredients, with at least 70% of the food produced by local land users (Lingu, 2023). In terms of environmental stewardship, Vama Buzăului is committed to conserving biodiversity, as demonstrated by the establishment of the Bison Valley Reserve in 2008. This reserve is dedicated to preserving and rewilding a sub-population of the European Bison (*Bison bonasus* L.), strengthening the region’s ecological integrity and enhancing the cultural and socio-economic aspects of the local communities. This initiative aligns proficiently with TMCL principles, where the interplay between natural and cultural elements is crucial (VBTIC, 2023). Table 1 represents the multifunctionality of Vama Buzăului based on its environmental, social and economic dimensions.

**Table 1:** Multifunctionality of the village of Vama Buzăului, Romania based on the environmental, social, and economic dimension

Dimension	Parameters	Examples in Vama Buzăului
Environmental	Habitat, biodiversity & agrobiodiversity functions; ecosystem services (regulating, provisioning, cultural), protected areas, landscape aesthetics, environmental quality as a basis for ecotourism.	Bison Valley Reserve for rewilding and conservation, improved waste management, preserved natural landscapes offering ecological functions and recreational opportunities.
Social	Cultural heritage & identity, community cohesion, preservation of local traditions, social and cultural services, learning/interpretation and cultural exchange.	Revitalized local traditions through festivals (e.g., Folk festival ‘Vama de la Mute’), the gastrolocal movement reinforcing culinary heritage; active community participation, community pride and recurring local festivals.
Economic	Local employment, livelihood diversification, infrastructure development (transport, connectivity, utilities), growth local value chains through local markets and entrepreneurial activities.	Creation of ecotourism-related jobs, increased economic opportunities, upgraded roads and enhanced public services that support local commerce and visitation.

Multifunctionality is used analytically to denote the demonstrable co-existence and co-production of multiple function bundles at landscape scale, not merely ‘more than one use’. This table provides a descriptive baseline of the co-existing function bundles observed/characteristic in Vama Buzăului as a TMCL system, to support interpretation of stakeholder perceptions and trade-offs

### Methods

This study utilizes a qualitative research methodology to examine local stakeholders’ perspectives regarding ecotourism in TMCLs. The qualitative approach comprehensively explores stakeholders’ subjective experiences, interpretations, and nuanced perceptions within their socio-cultural contexts.

Data were collected through semi-structured interviews conducted with twelve stakeholders from Vama Buzăului. In order to ensure participant anonymity and minimize bias, numerical identifiers were assigned to the participants, and all personal identifying details, such as names, ages, and genders, were excluded from the analysis and presentation of results. Table 2 shows the IDs assigned to the interviewees and their link with ecotourism.

**Table 2:** Interviewee IDs and link with ecotourism

Interviewee ID	Ecotourism link	Comment
IV1	Indirect	Resident
IV2	Direct	Accommodation owner
IV3	Direct	Gastrolocal owner
IV4	Indirect	Resident
IV5	Indirect	Resident
IV6	Direct	Administrative
IV7	Direct	Alternative form of accommodation
IV8	Indirect	Resident
IV9	Direct	Accommodation owner
IV10	Indirect	Resident
IV11	Indirect	Resident
IV12	Direct	Administrative

Interviews aimed to capture diverse viewpoints on ecotourism and its impacts in the TMCL context of Vama Buzăului. Conducted in Romanian language with the support of co-authors, interviews ensured clarity and authenticity. Transcripts were translated verbatim into English to maintain accuracy and preserve original meanings and contexts. Verbal consent was obtained from participants prior to commencing and recording interviews

Participants were selected via purposive sampling, a non-probability method allowing researcher discretion to include individuals deemed most relevant to the study objectives (Quinlan, et al., 2018) (Quinlan, et al., 2018). A comprehensive sample was taken to reflect the range of perspectives within the community, including both individuals directly engaged in ecotourism activities (such as entrepreneurs, administrative representatives, and those involved with conservation or gastrolocal initiatives) as well as those indirectly connected (residents). This ensured that the analysis captured a multifaceted representation of stakeholder experiences and perceptions relevant to ecotourism development in the TMCL. Thematic analysis was used to identify, analyze and report patterns within the interview transcripts, following a six-phase framework outlined by (Maguire & Delahunt, 2017). It includes becoming familiar with the data, generating initial codes, searching for themes, reviewing themes, defining themes, and writing up the findings (p. 3354). This method is selected for its wide use and systematic strengths in exploring the depth and structure of the data. The thematic analysis was performed using f4analyse software.

Themes were derived based on predetermined research objectives, with the data systematically indexed according to this framework to enable structured comparisons across stakeholder perspectives. The indexed data was organized into a matrix, where rows represented stakeholders and columns represented themes, facilitating identification and synthesis of patterns and divergences. The final analysis stage involved mapping and interpreting the data to elucidate key dimensions of the data. The themes were explicitly linked to the research objectives through interpretative mapping. This stage provided comprehensive insights, linking identified themes to our research objectives, broader theoretical and empirical contexts. This systematic, structured methodology ensures a rigorous and comprehensive analysis, highlighting recurring themes and contributing to a deeper understanding of stakeholder perceptions.

## Results

The analysis of interview transcripts based on the objectives resulted in the identification and extraction of five thematic areas, which are the positive and negative impacts of ecotourism on the socio-cultural, economic, and environmental dimensions of the site, the role of ecotourism in addressing land abandonment and outward migration, perceived benefits for stakeholders not directly involved in ecotourism, the role of the local government in ecotourism development and stakeholders perspectives of future requirements for ecotourism development. The extracted themes are summarized in Table 3. Each theme is then discussed in detail below and the results are interpreted with respect to the conceptualization Vama Buzăului as a TMCL.

**Table 3:** Key themes from the thematic analysis

Theme	Positive Outcomes	Challenges/Concerns
Socio-cultural impacts	Enhanced community pride and revitalized traditions. Increased cultural exchange and social cohesion.	Risk of cultural commodification. Disruptions due to overcrowding.
Economic impacts	Growth of local businesses and job creation. Improved incomes and upgraded infrastructure.	Rising land prices. Unequal benefit distribution among community members.
Environmental impacts	Improved waste management and environmental awareness. Successful conservation initiatives (e.g., Bison Reserve).	Increased litter and traffic congestion. Reduced communal grazing areas.
Land abandonment and outward migration	Incentivizes youth retention. Repurposes idle land for ecotourism ventures.	Potential alteration of traditional land-use patterns by outsider investments.
Benefits to non-tourism stakeholders	Indirect benefits through improved public infrastructure and expanded market opportunities.	Perceived marginalization of those not directly involved in ecotourism. Increased cost of living and property prices.
Role of local government	Effective administrative support and infrastructural investments. Facilitation of bureaucratic processes.	Insufficient direct financial support. Need for enhanced management of ecotourism related impacts.
Future requirements	Demand for diversified attractions. Need for financial incentives such as micro-loans and capacity-building.	Concerns about preserving cultural authenticity amid ecotourism expansion.

Since, interviewees sometimes used ‘tourism’ colloquially to denote ecotourism, our interpretation focuses on the eco-certified ecotourism initiative as the institutional object of analysis while acknowledging that some pressures (e.g., traffic/crowding) may reflect broader visitation dynamics. While many of these impact categories are widely reported in tourism–community research (e.g., sustainability–resilience and community livability lenses), TMCL-specific evidence interpreted through a regenerative lens remains limited, which is where our contribution lies (Giang & Caldicott, 2022; Muschter, et al., 2021).

### Local Stakeholder Perceptions on Ecotourism’s Impacts (Positive and Negative)

#### Socio-Cultural Impacts

Community members in Vama Buzăului generally viewed the influx of visitors positively for its social and cultural benefits. Several interviewees expressed that tourism has permeated new energy into local traditions and festivals. *“It’s nice to see new faces and know that people appreciate our town,”* mentioned one interviewee (IV1), noting that events like the annual folk music festival “Vama de la Munte” attracted unprecedented attendance from tourists, especially after the COVID pandemic. This increased external interest was perceived to bolster local pride and encourage the preservation of cultural heritage. For example, an interviewee (IV3) mentioned *“For me it is not a job, I am neither a chef, nor a waiter, nor a patron, I am a host. This is how I see the concept* [of ecotourism] *and I do it with passion and pride”* suggesting that what was once everyday life is now recognized as something of value. Another interviewee (IV12) also emphasized the positive recognition of local traditions: *“I love that our traditions are being noticed.”* Similarly, another interviewee (IV5) added, “*People come from far, as far as Bucharest on weekends to enjoy our traditional food & recipes.”* Some stakeholders also credited tourism with fostering social cohesion, as neighbors collaborate to host visitors and share advice. One interviewee (IV4) mentioned that ecotourism has *“connected us as a community because I see that people help each other and ask for advice* […] *this brings added value to our community.”* These accounts indicate a sense of empowerment and validation of local culture through tourism engagement. However, a few stakeholders voiced concerns about potential socio-cultural downsides. The most commonly cited issue was the inconvenience caused by crowding during peak tourist visit times like weekends. *“Sometimes there can be a lot of tourists and it’s hard to go about our day* […] *running into crowds everywhere,”* mentioned one interviewee (IV1), illustrating how an influx of visitors can disrupt daily routines. Another interviewee (IV5) worried that cultural authenticity might be compromised as traditions become commodified for tourist consumption. *“Sometimes I feel like our culture is being sold as tourist attractions,”* they noted, *“but it is the way of the world.”* Similarly, another interviewee (IV2) also observed that crowding effects community members, *“those who are not in charge of tourism are a bit against tourists, it bothers them that the commune is crowded.”* Such comments reflect a cautious awareness that while tourism brings recognition, it also risks diluting the intimate, authentic character of the TMCL. Overall, socio-cultural perceptions were positive, highlighting increased community pride and cultural vitality tempered by minority concerns about overcrowding and maintaining authenticity.

These accounts indicate a sense of empowerment and validation of local culture through ecotourism engagement. The revival of festivals and the gastrolocal movement can also be interpreted as regenerative practices, where ecotourism strengthens cultural vitality rather than merely sustaining it.

#### Economic Impacts

Nearly all local stakeholders acknowledged economic benefits from ecotourism development in the community. Stakeholders pointed to new livelihood opportunities emerging along with tourism growth. *“Local businesses like the gastrolocals and shops are making more money,”* mentioned one interviewee (IV1), adding that *“there are more job opportunities, especially for young people”*. This perception was echoed by multiple participants who observed employment opportunities in new guesthouses, shops and other ecotourism related services that have risen up to cater the visitors. The opening of gastrolocal points which serve locally grown traditional food and other gastronomic products was repeatedly mentioned as an example of how ecotourism stimulates the local economy beyond formal ecotourism jobs. As mentioned by an interviewee (IV3), *“they* [tourists] *come to experiment and see what the gastrolocal is all about and the flow of tourists in the last 4 years has increased tremendously”*. Farmers, too, have benefited indirectly by selling more produce and dairy to these businesses. As one interviewee (IV8) mentioned, *“even if you’re not in the tourism business, you still see benefits. For example, as a farmer, I sell cheese and vegetables to gastrolocals* […] *everyone in the community benefits from it”*. In addition to income generation, stakeholders highlighted improvements in infrastructure attributable to ecotourism. They noted that roads have been upgraded, a public Wi-Fi network has been installed, and transportation links to the nearest city have been made more reliable, changes which *“make it easier to get around”* for community members and visitors alike. Such investments were appreciated as they improved the quality of life for residents. As mentioned by an interviewee (IV1), *“We have free Wi-Fi, regular bus to the city, electricity because people* [tourists] *come from* [urban] *places like Bucharest and they want these things”*.

Despite these broad gains, the economic upsides were not perceived to reach everyone equally. The unequal distribution of benefits was a recurrent theme. A few stakeholders pointed out that families directly involved ecotourism businesses enjoy most of the profits, whereas others on the periphery feel left out. *“Not everyone has equal benefits* […] *Some locals would rather have industries or factories built here”* one interviewee (IV1) mentioned. Another interviewee (IV8) mentioned, *“if a factory was opened in the area, three would be more jobs. It is true there would be more work for all”*. These remarks underline a lingering perception among a minority that ecotourism, while lucrative for some, might not be the appropriate solution for all community members. Nevertheless, no respondents reported any severe negative economic impacts, such as loss of livelihoods; instead, the concern was about relative inclusivity. One nuanced economic impact noted was the rising land price in the area. As an interviewee (IV7) mentioned, *“since the past 4,5 years the value of land in Vama has felt an increase”*. This trend can benefit landowners but may make it costlier for locals (especially youth) to acquire property. Local stakeholders largely credit ecotourism with revitalizing their economy creating jobs, increasing household incomes, and prompting infrastructural improvements while recognizing the need to broaden participation to share economic benefits equally.

Stakeholders not directly involved in ecotourism too, have benefited indirectly by selling more products and dairy to these businesses. This integration of traditional livelihoods into new markets exemplifies a regenerative dynamic, as it revitalizes agricultural and traditional practices (like wood working and crafts) while creating new opportunities.

#### Environmental Impacts

Stakeholder perspectives on ecotourism’s environmental impact were ambivalent, with relatively fewer positive observations compared to the socio-economic dimension. On the one hand, community pride in the area’s natural assets has grown alongside ecotourism. Locals were proud that Vama Buzăului is now known as *“where the bison reserve* [and] *Urlătoarea waterfall* [are]*,”* as one interviewee (IV4) mentioned. Some stakeholders believed that ecotourism has prompted better environmental management by authorities and the community. As an interviewee mentioned (IV6), *“From a point of view, the reservation has given the Vama Buzăului commune a big mouthful of oxygen.”* Another interviewee mentioned (IV4) *“waste collection services have improved after ecotourism took off, new trash bins are installed, and garbage disposal has become more regular and waste separation is done for improved recycling”*. There were even anecdotes of environmentally responsible behavior among visitors, one interviewee (IV6) mentioned seeing tourists carry their litter out of the forest and use the first available trash can, striving to *“keep* [the area] *just as they found it”*. On the other hand, several interviewees raised concerns about negative environmental pressures resulting from increased visitation. Increased waste was the most frequently mentioned issue, *“automatically there will be waste or all kinds of extra debris when more people enter the community”*, one interviewee (IV10) mentioned. Littering in the bison reserve and along hiking trails was cited as an occasional problem caused by some visitors. *“Sometimes I find litter near the river or in the fields, and that upsets me. We take care of this land, it’s our home.”*, (IV10). Stakeholders noted heavier vehicle traffic on weekends, leading to noise, air pollution, and even safety hazards on narrow rural roads. *“There are many cars, and the streets are narrow*, [it is] *a little dangerous to walk. There are kids playing around, and animals too,”* one interviewee (IV8) mentioned. Another interviewee (IV12) added *“Now, we have people coming in every weekend, cars parked everywhere.”* Some stakeholders also discussed broader land use impacts. The establishment of the bison reservation itself was a point in question for a minority as about 10 hectares of former communal grazing land were fenced off for the reserve, reducing pasture available to villagers’ livestock. *“People were less receptive* [to tourism at first] *because that area* [present Bison reserve] *was used for grazing animals,”* an interviewee (IV6) mentioned, noting that any loss of traditional grazing area *“does not always sound good”* to those dependent on livestock. This highlights an important environmental trade off inherent in the ecotourism project that is sometimes dedicated conservation land can constrain other uses. Despite these concerns, it is noteworthy that no interviewee perceived any catastrophic environmental damage from ecotourism rather the concerns were manageable issues like litter, congestion, and land use changes. Instead, initiatives like the bison reserve and improved waste management indicate that ecotourism can act as a regenerative force, enhancing ecosystem health while instilling ecological pride among residents. The stakeholder’s overall point of view was that ecotourism must continue to prioritize environmental care to ensure the long-term sustainability of the TMCL.

### Ecotourism’s Role in Addressing Land Abandonment and Outward Migration

Most stakeholders believed that ecotourism’s growth has directly contributed to retaining the local population, especially youth who might otherwise leave this rural area. One interviewee (IV12) shared, *“Yes, of course* [ecotourism has helped]*. There are a lot of young people who have taken the initiative to return to Vama because they have the opportunity to work in their home village and earn some money.”* This statement exemplifies a common perception that new ecotourism-related enterprises (guesthouses, gastrolocals, handicrafts, etc.) are persuading back some who had left and dissuading others from leaving in the first place. Multiple respondents provided concrete anecdotes of this trend. For instance, an interviewee (IV5) mentioned that her grandson, who previously commuted to Brașov for weekend work, *“now helps in a gastrolocal nearby and is satisfied.”* Similarly, another interviewee (IV10) mentioned, *“I like that it’s keeping people here. I see young folks staying, finding work at Gastrolocals and guesthouses or selling homemade products instead of running off to Braşov or Bucharest.”* These perspectives illustrate how ecotourism creates local jobs that allow people to make a living while remaining in the community. As a result, interviewees widely credited ecotourism for strengthening family ties and social structures that might have been undermined by youth out-migration in the past.

Regarding land abandonment, stakeholders linked ecotourism with a more active use of local land and properties. There was a shared notion that formerly idle or underutilized land is now seen as an opportunity. *“For the young people of Vama Buzăului, it is an opportunity, like others, to open rooms for rent”* mentioned an interviewee (IV9), implying that instead of leaving land vacant or selling it off cheaply, families are investing in small-scale tourist accommodation. Even those community members not opening guesthouses often lease their fields or homesteads to neighbors who need land, ensuring it *“is not empty.”* This indicates that the prospect of ecotourism is incentivizing locals to maintain ownership and productive use of their land rather than abandoning it entirely. As mentioned by an interviewee (IV4), “*This* [ecotourism] *thing keeps us here because you have something to do* […] *if you want to get down to business, you have something to do.”*

While the overall picture was optimistic, a few nuanced perspectives emerged. One of the interviewees (IV12) mentioned that more people especially younger families were staying in Vama Buzăului because of the new opportunities and that *“land isn’t being abandoned.” “But,”* he cautioned, *“it’s not always locals who are buying it. It’s outsiders, and that changes things.”* This observation reveals an underlying complexity that is the growing interest in the area which has attracted outside investors or new residents who purchase the property. Such changes can have mixed implications, for instance abandoned homes are being occupied and renovated, injecting capital into the village but the social fabric and traditional land use patterns may shift if external actors become major landowners. However, despite this note of caution, no interviewee suggested that tourism is causing people to leave. Instead, the worst-case scenario expressed was that ecotourism alone might not be sufficient to keep everyone from migrating outwards due to lack of economic opportunities, particularly so if they do not feel included in the benefits.

Families are investing in small-scale tourist accommodation. This reactivation of previously idle land reflects regenerative land use, where ecotourism contributes to reversing abandonment and strengthening socio-ecological resilience.

### Benefit Distribution

The study also explored whether the benefits of ecotourism extend to ‘non-tourism” stakeholders that is those not directly employed in the tourism sector. According to local stakeholders, the indirect benefits of tourism are felt across the community, although to varying degrees. A strong consensus emerged that improved public infrastructure driven by ecotourism serves all residents. For example, better roads, enhanced public transport, and cleaner public spaces were repeatedly cited as “*community-wide gains”*. As one interviewee (IV12) put it, *“The improved roads are used by all and not just those in tourism. The public spaces are nicer now too, and there are more recreational activities* […] *everyone in the community benefits from it.”* This highlights that investments initiated by ecotourism have a ripple effect, increasing accessibility and quality of life for the entire community and not just tourists or tourism businesses.

Another commonly mentioned indirect benefit was the expansion of markets for local products. Interviewees mentioned small-scale farmers selling more eggs, milk, cheese, and vegetables to nearby gastrolocals or at the weekend farmers’ market because visitor demand supplements the usual local consumption. As mentioned by an interviewee (IV10), *“Even if you do not own a guesthouse or a restaurant, you can still benefit* […] *farmers sell more produce, craftsmen like me sell more handmade goods* […] *and the roads are better! That helps everyone, not just the tourists.”* Testimonies such as these demonstrate a widely perceived integration of ecotourism into the local value chain. Ecotourism creates additional income streams for residents who engage in traditional livelihoods (farming, animal husbandry, crafts), reinforcing those livelihoods rather than replacing them.

Ecotourism was also credited with intangible communal benefits. By bringing outside visitors, tourism has subtly broadened locals’ horizons and created opportunities for cultural exchange. Exemplifying this sentiment one interviewee (IV9) mentioned, “*We had guests, my father had tourists from Scotland, tourists from England, from Italy, so also foreign tourists. And I liked it . From my point of view, I guess, interacting with other people from other places, even foreign people, you learn from them ”*. Additionally, organizing tourism activities (like village tours or folk music festivals) has required cooperation among villagers, which some said has improved community relationships and collective action. One stakeholder (IV2) mentioned that even those not in tourism have become more involved in community events (like the Vama de La Munte or cleaning campaigns), partly because these events have grown in prominence due to tourism.

Despite these positive accounts, a few stakeholders pointed out that indirect or passive benefits can only go so far. Not everyone felt personally touched by ecotourism’s prosperity. *“Some benefit, but not everyone,”* mentioned one interviewee (IV5), acknowledging that households with existing resources to engage with tourists see more gains than those who do not. The general sentiment was that anyone who wanted to participate could find a way to benefit, even if indirectly but if a person chose not to get involved, they might feel relatively left out. As mentioned by an interviewee (IV11), “*If you’re just living here, working a normal job, all you see is the prices going up, the village getting busier.”* Notably, there was no indication of social conflict or resentment between those in ecotourism and those out of it. Instead, there was a recognition that ecotourism is a new opportunity that some seize more actively than others. Some stakeholders mentioned discussions within their families or among neighbors about starting some modest ecotourism venture (such as opening a small gastrolocal eatery or building a couple of rooms for rent) to take advantage of the growing visitor numbers.

### Local Government’s Role in Ecotourism Development

Local government institutions particularly the Vama Buzăului municipal administration emerged as key actor in the ecotourism initiative, and stakeholders’ perceptions of their support were generally favorable. Many interviewees acknowledged that the mayor and local council have proactively facilitated ecotourism development. *“The local government has helped the community right from the start in all manners,”* said one interviewee (IV5), *“like applying* [for] *funding or preparing documents to get involved in ecotourism.”* Multiple participants mentioned that the local administration provided guidance in navigating bureaucratic procedures such as obtaining permits, certifications, or accessing development grants for community members who started accommodations or established gastrolocal points or other ecotourism businesses. This administrative assistance was highly valued. As exemplified by an interviewee (IV8), *“the council helps with the paperwork* […] *there is a lot of governmental process to complete to open the business, and* [they] *help.”* Such hands-on support can lower barriers to entry, enabling more locals to participate in ecotourism. Additionally, the local government’s strategic investments and partnerships were highlighted by the stakeholders. Interviewees credited the local government for starting infrastructure improvements such as roads, signage, waste management, and internet connectivity that have made the village more attractive and accessible to tourists. Brașov County also provided an 80-hectare forest land to the commune in 2008 for free use in conservation and ecotourism. This multi-level governmental backing has been crucial in establishing Vama Buzăului’s reputation as an eco-certified destination. From the community’s perspective, these efforts translate into concrete support, *“Those who run this municipality* […] *especially the mayor* […] *I have all the respect,”* mentioned an interviewee (IV1) expressing trust that local leaders are steering the community in a positive direction.

However, perceptions of government support were not unanimously positive. A few interviewees reported little to no direct support for their initiatives, especially financially. *“No* [support]*. Just my own funds* […] *no outside* [help]*, I funded my own business”* one interviewee (IV9) mentioned, implying that while general infrastructure was improved, he did not receive any grants or subsidies to start his business. Some stakeholders felt the authorities could do more to manage flows to reduce problems like traffic and litter. Such comment can indicate an expectation for continuous government involvement in mitigating ecotourism’s adverse impacts as they arise. The local government is seen by local stakeholders as an active partner in ecotourism development, providing institutional support that ranges from bureaucratic facilitation to infrastructure provision.

### Future Requirements for Sustainable Tourism Development

When discussing the future, local stakeholders articulated clear aspirations as well as concerns for ecotourism development. A dominant response to future requirements was to diversify and expand the ecotourism offerings in Vama Buzăului. Many interviewees noted that the current ecotourism is mostly limited to the Bison Reserve (Valea Zimbrilor) and the Urlătoarea waterfall. While these two attractions have put the commune on the map, residents worry that relying on only a few sites could limit growth or limit repeat visits. *“I would like to see more activities for visitors. Right now, they come for the bison and the waterfall, but after that, they do not have much to do,”* one interviewee (IV10) mentioned. Others echoed that sentiment, suggesting the development of additional tourist attractions such as cultural trails, historical sites, agritourism experiences on local farms, or adventure activities to encourage longer stays and repeat visits. For instance, (IV2) mentioned, *“for the tourist who comes to Vama Buzăului […] you need more places to visit […] bike trails […] all sorts of activities […] to stay as many days as possible.”* Similarly, (IV7) added, “*we are not limited to having only the bison reservation, the Urlătoarea waterfall […] the tourist […] may want more things.”* Another commonly cited requirement was financial assistance or incentives for local entrepreneurs. *“It would be more encouraging if* [the authorities] *provided, you know, small loans interest-free or with low interest to start* [a tourism] *business”* mentioned an interviewee (IV8). The idea of micro-finance or grant programs specifically aimed at rural ecotourism development was seen as a catalyst that could help families who lack the capital to join the ecotourism sector. This also indicates a desire of the broader community of being involved in the ecotourism sector. Alongside funding, training in hospitality and marketing was mentioned as a future need, especially if Vama Buzăului is to attract more international tourists.

Preserving the intrinsic characteristics and structure of the TMCL amid development was another recurring theme in these future visions. Stakeholders are proud of Vama Buzăului’s natural beauty and cultural traditions, and want tourism growth to be balanced with conservation. As mentioned by an interviewee (IV12), *“I would like for Vama Buzăului to develop more* [tourist attractions] *so that we are not limited to only the bison and the waterfall, but sometimes I worry if in the future our culture and traditions will be modernized* [too much] *to bring more tourists.”* This quote captures the general dual aspirations to advance and innovate in ecotourism, yet not at the expense of the tangible and intangible heritage that makes the area unique. Stakeholders mentioned that any new attractions should be authentic and rooted in local culture or nature, for example workshops reviving old crafts, organizing genuine traditional festivals for tourists, or creating nature education centers and guided hiking tours. The interviews conveyed a desire for participatory planning: people feel their intimate knowledge of the land and culture is an asset that should guide development decisions, ensuring ecotourism remains appropriate to the TMCL context.

## Discussion

Our findings from Vama Buzăului underscore the high importance of engaging local stakeholder perspectives in ecotourism development, particularly within TMCLs. The results provide a nuanced account of how residents perceive the benefits and challenges of ecotourism in TMCLs. The local perception in Vama Buzăului reveals how bottom-up involvement can translate the abstract promise of ecotourism into tangible socio-economic gains at local level. In line with previous findings (Snyman, 2016; Samal & Dash, 2023) that private ecotourism enterprises can foster local socio-economic development when communities are involved, Vama Buzăului’s experience shows a collaborative model in practice: the local government, together with residents, actively shaped the ecotourism initiative to align with community needs. By documenting local stakeholder’s enthusiasm for improved infrastructure, cultural revival, and livelihood opportunities, our study reinforces arguments in the ecotourism literature that empowering local stakeholders leads to more collectively desired outcomes and can facilitate sustainability (Anindhita, et al., 2024). For instance, (Ruiz-Ballesteros, 2011) observed that involving communities in ecotourism can build social cohesion and resilience. This dynamic is evident in Vama Buzăului, where interviewees described greater community cooperation and pride stemming from the development of ecotourism in the area. The participatory ethos in Vama Buzăului’s planning aligns with emphasis on stakeholder collaboration as a cornerstone of sustainable ecotourism (Wondirad, et al., 2020). Thus, the study also provides empirical support for theoretical frameworks that advocate inclusive governance in ecotourism development within traditional cultural landscapes.

We use ‘regenerative tourism’ as an interpretive lens to assess whether ecotourism strengthens local stewardship, cultural vitality, and the community’s capability to sustain TMCL multifunctionality over time, rather than only minimizing impacts. Viewed through a regenerative tourism lens, these findings suggest that ecotourism in TMCLs can move beyond sustaining existing systems to actively revitalizing them. Cultural traditions are not merely maintained but renewed; landscapes are not only conserved but reactivated for multifunctional purposes; and community relations are strengthened in ways that enhance long term socio-ecological resilience (Mang & Reed, 2012; Bellato, et al., 2022).

### Socio-Cultural Impacts of Ecotourism

The results of this study reflect the dual nature of ecotourism’s socio-cultural impacts in Vama Buzăului. On the one side, the community’s positive outcomes like revitalized traditions, heightened cultural pride, and strengthened social fabric are examples of how ecotourism can serve as a tool for cultural conservation and rural revitalization. (Mondino & Beery, 2019) noted that ecotourism can act as a learning tool for sustainable development, helping communities revalue their cultural and natural heritage in countries like Italy. Similarly, our findings show that locals increasingly cherish their heritage (festivals, crafts, local gastronomy) as they see visitors appreciating these assets. At the same time, stakeholders’ concerns about cultural commodification and authenticity loss align with existing literature (Kneafsey, 2000; Koens, et al., 2009; Doan, 2010) in rural ecotourism. It should be noted that the commercialization of the stakeholder’s daily life to meet tourist expectations can create tension between economic interests and cultural integrity. This contrasts with cases where outside operators dominate tourism, and locals feel alienated (Koens, et al., 2009).

In regenerative terms, this cultural revitalization illustrates how heritage can be dynamically reinterpreted for contemporary relevance (Sharma & Thama, 2023). The gastrolocal movement, artisanal crafts and traditional festivals embody regenerative practices that embed cultural continuity within evolving economic and social context (Bellato, et al., 2022). This dynamic reflects the capacity of ecotourism in TMCLs to strengthen cultural identity while fostering intergenerational continuity.

### Economic Impacts and Distribution of Benefits

Economically, this case study reaffirms that ecotourism can generate significant local income and employment, as reported in the literature (Doan, 2010; Mudasir, et al., 2020). However, a critical insight from this study is the issue of uneven benefit distribution. Statements such as “*not everyone has equal benefits”* shows the local perception that ecotourism’s gains often accrue disproportionately to those with resources or proximity to the attractions, potentially exacerbating local inequalities if left unaddressed. This finding underscores the need for mechanisms to broaden participation and develop benefit sharing schemes to ensure inclusive development.

From a regenerative perspective, the results highlight the capacity of ecotourism to generate new opportunities and stimulate local entrepreneurship. The regenerative lens emphasizes circularity and inclusivity in economic systems, where the gains derived from ecotourism are circulated broadly to reinforce local livelihoods rather than being concentrated among a small group of stakeholders (Bellato, et al., 2022). This kind of approach contributes not only to resilience but also socio-economic sustainability in the long term (Miedes-Ugarte & Flores-Ruiz, 2025).

### Environmental Impacts

Residents noted improvements in waste management and a culture of cleanliness that aligns with ecotourism principles. This suggests that when communities embrace ecotourism, they may also internalize stronger pro-environment behaviors which may be indicative of ecological consciousness rising in host communities (Wondirad, et al., 2020; Samal & Dash, 2023). Moreover, the pride locals feel in their natural and cultural heritage indicates a sense of environmental stewardship ecotourism fosters (Smithwick, et al., 2023) (Smithwick, et al., 2023). However, the community’s concerns about litter, traffic, and the loss of grazing land illustrate the real challenges of balancing increased human activity with environmental protection. These micro-level issues mirror larger debates in ecotourism literature about carrying capacity and the risk of degrading natural resources as ecotourism grows in scale (Wondirad, et al., 2020; Samal & Dash, 2023).

Enhanced waste management services and the conservation of the European Bison exemplify a shift towards regenerative practices, in which ecotourism not only minimizes ecological harm but actively contributes to ecosystem restoration and stewardship (Smithwick, et al., 2023; Buckton, et al., 2023). Regenerative tourism thereby reframes environmental protection as a process of enrichment and renewal, aligning ecological sustainability with community wellbeing (Mang & Reed, 2012).

### Ecotourism, Land Abandonment, and Outward Migration

A particularly novel contribution of this case study is its examination of ecotourism’s role in mitigating land abandonment and outward migration. Our findings provide empirical evidence that ecotourism can help address the problem of rural outmigration and land abandonment by creating attractive local opportunities. This supports anecdotal assertions in policy discourse that ecotourism is a promising strategy to combat depopulation (Martos et al., 2024). In Vama Buzăului, the community’s testimonies indicated a stabilization (even a slight increase) of the resident population, especially among working-age youth. Similarly, the study shows evidence of youth returning from cities and families repurposing land for ecotourism rather than selling it pointing to a demographic and land-use turnaround partly facilitated by ecotourism. This is a significant insight for TMCLs as it demonstrates that multifunctional land use (combining traditional farming with new ecotourism functions) can enhance the viability of rural communities, as suggested by Zerbe (2025).

Furthermore, the findings also reveal that while ecotourism reduced out-migration by providing jobs, it simultaneously increased outside investment (people from outside the area buying properties, drawn by the area’s appeal). This reflects a form of rural gentrification or amenity migration, a phenomenon where affluent outsiders move into scenic rural areas (Gustafson, 2002) which can have mixed effects on local social structures. Responses from interviewees show that local stakeholders are aware and somewhat weary of this phenomenon. These findings contribute to a more nuanced understanding of ecotourism’s complex impact. However, planners should monitor and manage the implications of new migration patterns, for example, by encouraging local land owners to retain the land and preventing speculative land grabs.

Regenerative tourism highlights the abovementioned transformations as evidence of renewed vitality (Bellato, et al., 2022). By reactivating abandoned land and strengthening local ties, ecotourism demonstrates regenerative potential to reverse socio-economic decline in TMCLs.

### Governance and the Role of Local Government

Similarly, the case of Vama Buzăului offers theoretical insights and practical implications for ecotourism in TMCLs. Theoretically, it exemplifies the concept of multifunctionality as the landscape is simultaneously a place of agriculture, habitation, cultural heritage, and ecotourism. The ability of the community to integrate a new function (ecotourism) without displacing the traditional ones (farming, grazing, local festivals) speaks to the resilience and adaptability of TMCLs. It provides a living example of what Zerbe (2025) describes as merging tradition and innovation for a sustainable future. Local stakeholders effectively became agents of this merge, ensuring that ecotourism development is embedded in the local context (for example, gastrolocal restaurants built on traditional cuisine and local ingredients) rather than imposed from outside. Additionally, the social outcomes like enhanced community cooperation support social exchange and empowerment facilitated by ecotourism in TMCLs.

When residents perceive the exchange with tourists to be beneficial, they respond with more significant support and engagement. Practically, the findings suggest recommendations for ecotourism governance in TMCLs and similar rural areas. Our governance implications are deliberately constrained to what the interviews support that is stakeholders emphasized municipal facilitation (paperwork, infrastructure, enabling conditions) and expectations for fairer benefit sharing rather than documenting a formal participatory council architecture.

Firstly, inclusive planning frameworks are crucial. Early and ongoing involvement of local people in decision making is something other regions could emulate by setting up participatory councils and committees where community members can voice ideas and concerns. As our study shows, locals bring invaluable perspectives on appropriate development and where sensitive limits lie (such as how much commercialization of culture is acceptable). Planners and policymakers in rural development should treat local knowledge as a central input, not an afterthought. Secondly, the role of local government is pivotal in catalyzing ecotourism, so building strong public-community partnerships is crucial. In this case, the local authorities provided infrastructure and helped knit together a network of support which ensured that ecotourism was backed by policy (eco-certification, funding, land grants) beyond the destination’s limited resources. Thirdly, mechanisms to spread benefits widely should be part of ecotourism policy.

In regenerative tourism, governance is recognized as a key enabler of systematic transformation (Bellato, et al., 2022; Smithwick, et al., 2023; Sharma & Thama, 2023). Local governments’ facilitative role in Vama Buzăului suggests potential to formalize regenerative pathways by embedding participatory governance, infrastructure development and support schemes that promote socio-cultural renewal and ecosystem health simultaneously.

### Future Pathways: Towards Regenerative Ecotourism

Vama Buzăului’s locals highlighted indirect benefit channels (infrastructure, markets for produce). Formalizing these channels can maximize equity. For example, destination planners could establish local procurement policies (so that accommodations source a percentage of supplies from nearby farms and artisans) or community development funds where a portion of ecotourism revenue is reinvested into public services like schools and medical clinics. These steps ensure that even those not directly in ecotourism feel a positive impact, increasing overall community support. Similarly, findings indicate a need for capacity building and financial support to enable interested locals to join the ecotourism sector. Training programs in hospitality, language skills, or digital marketing would empower willing community members to start businesses or improve existing ones, addressing some stakeholder’s hesitation about inequal benefit sharing. Credit or grant schemes which can be funded through local government programs or NGOs, could lower the barrier for low-income families interested in joining ecotourism as service providers. This would operationalize the community’s own vision of widespread grassroots entrepreneurship.

In this way, this case study contributes to the growing body of knowledge on ecotourism in traditional cultural landscapes by illuminating how local stakeholders perceive and influence the trajectory of sustainable development. The conceptual and empirical dimensions highlight the potential of TMCLs as integrated socio-ecological systems capable of contributing meaningfully to the broader global agenda of sustainable development. It confirms that integrating local perspectives is not just a matter of equity but enhances the effectiveness and resilience of ecotourism initiatives. The study’s insights into sociocultural revitalization, economic inclusion, demographic stabilization, and governance provide a grounded example for comparison with other TMCLs pursuing ecotourism as a development strategy. It highlights that while challenges such as benefit distribution and cultural commodification exist, they can be managed through community empowerment and adaptive governance. Vama Buzăului stands as an encouraging example of a TMCL leveraging its natural and cultural heritage through ecotourism for community development.

However, it is crucial to avoid replicating the growth-driven development paths that compromised sustainability in rural tourism destinations in Western Europe during the 1970s. Previous research (Ferguson, 2015; Panzer-Krause, 2019) indicates that purely expansionist approaches prioritizing continuous increase in visitor numbers and infrastructure often led to sociocultural commodification, ecological degradation and weakening of local governance structures. As (Sharpley, 2009) and (Saarinen, 2006) argue, when tourism development privileges economic metrics over community well-being and environmental stewardship, it can erode the very cultural and ecological assets upon which rural tourism depends. In the context of Vama Buzăului, where local stakeholder engagement and the integration of ecotourism with cultural heritage have so far supported sustainable outcomes, it is critical to reinforce development strategies that move beyond simple expansion.

Adopting a regenerative development framework allow Vama Buzăului to avoid growth driven models of mass tourism and instead pursue quality driven, place based innovation (Sharma & Thama, 2023). Practical implementations could include channeling ecotourism revenue and volunteer initiatives into habitat restoration, cultural heritage projects, afforestation programs, wildlife monitoring, and traditional craft workshops. Such activities transform ecotourism into a mechanism for enriching local ecosystems and preserving cultural traditions. To clarify the underlying mechanisms, we synthesize the empirically derived pathways across the socio-cultural, economic, environmental, and governance dimensions into a concise regenerative ecotourism development framework. Taken together, the results point to a small set of interlinked pathways through which ecotourism can become regenerative in TMCLs. Socio-cultural revitalization is reflected in renewed community pride and re-activated living traditions (festivals, gastronomy, place identity), while economic benefits extend beyond ecotourism operators through locally embedded value-chain linkages that connect accommodations and gastrolocals with farming and crafts yet remain contingent on perceived fairness in benefit distribution. In parallel, improved waste management and heightened recognition of conservation assets indicate emerging ecological stewardship, but stakeholders also highlight manageable pressures (litter, congestion, and land-use trade-offs), underscoring that enabling governance, municipal facilitation, infrastructure, and access to training/finance remains a necessary condition for maintaining multifunctionality over time. A careful shift from a quantity-driven to a quality-driven development model is thus essential for safeguarding the long-term sustainability of Vama Buzăului and similar TMCLs.

## Conclusion

This study examined local stakeholder perspectives on the multifaceted impacts of ecotourism within the TMCL of Vama Buzăului, Romania. Analysis revealed that ecotourism has fostered community cohesion, cultural revitalization, and economic growth through job creation, improved infrastructure, and innovative initiatives such as the gastrolocal movement. At the same time, stakeholders expressed concerns regarding cultural commodification, rising land prices, uneven benefit distribution, and environmental challenges such as increased waste and traffic congestion. The findings contribute to the existing body of knowledge by addressing a significant gap in the literature on ecotourism in TMCLs.

The results further suggest that while ecotourism can mitigate issues of land abandonment and outward migration, its sustainability depends on continuous policy support and adaptive management strategies that balance growth with the preservation of cultural authenticity, environmental integrity and contribute to regenerative socio-ecological outcomes. Practically, these insights offer valuable implications for policymakers and practitioners by highlighting the critical role of local government in facilitating infrastructure improvements and providing administrative support. Theoretically, the study reinforces that sustainable development in TMCLs requires a nuanced integration of traditional practices with modern ecotourism dynamics, ensuring that benefits are equally distributed among all community members.

Framing the results through a regenerative perspective further enriches the findings. This case is among the first to empirically interpret ecotourism in a TMCL through a regenerative lens highlighting pathways for policy and practice. Ecotourism in Vama Buzăului has not only sustained but actively revitalized socio-ecological systems. Cultural traditions such as festivals and gastronomy are reinterpreted for contemporary relevance, abandoned land is reactivated for ecotourism and multifunctional livelihoods and ecological stewardship is strengthened through initiatives like the bison reserve. These processes embody regenerative principles, where tourism produces net positive cultural, economic and environmental outcomes rather than merely reducing harm (Mang & Reed, 2012; Bellato et al., 2022).

These insights further demonstrate that the concept of TMCLs aligns closely with the Sustainable Development Goals (SDGs) objectives, offering a holistic framework for place-based sustainability. TMCLs inherently support SDG 15 (Life on Land) through biodiversity conservation rooted in traditional ecological practices. At the same time, their emphasis on cultural continuity and community resilience contributes to SDG 11 (Sustainable Cities and Communities). The multifunctionality of TMCLs linking agriculture, cultural heritage, and ecotourism advances SDG 8 (Decent Work and Economic Growth) and SDG 12 (Responsible Consumption and Production). The empirical findings from Vama Buzăului reinforce these linkages, illustrating how ecotourism within TMCLs can contribute to demographic stabilization through income generating activities (SDG 1: No poverty), foster environmental consciousness (SDG 13: Climate action), and enhance participatory governance (SDG 16: Peace justice and strong institutions) and multi-level collaboration (SDG 17: Partnership for goals).

Nevertheless, it is important to acknowledge certain limitations. The study’s qualitative approach, which relied on semi-structured interviews with a relatively small sample size, may restrict the generalizability of the findings. Accordingly, findings are analytically transferable rather than statistically generalizable, offering a grounded basis for comparison with other TMCLs and for hypothesis-building on regenerative pathways. Additionally, the cross-sectional nature of the research does not allow an evaluation of ongoing trends or the lasting effects of ecotourism on local socio-economic conditions, cultural and traditional aspects, and environmental stewardship; instead, it creates a foundation for future research. Future research should adopt longitudinal designs to better capture evolving stakeholder perceptions and long-term sustainability outcomes associated with ecotourism development in TMCLs. Comparative research involving multiple TMCL sites could provide deeper insights into context-specific factors influencing ecotourism’s success and challenges, thus informing more universally applicable policy frameworks. Further studies might also integrate mixed-methods approaches, combining qualitative and quantitative analyses.

Thus, Vama Buzăului exemplifies how ecotourism can foster sustainable regional development while preserving traditional cultural landscapes when effectively managed and embedded within local governance and community participation frameworks. Continued emphasis on inclusive stakeholder engagement and adaptive governance mechanisms is essential to ensure that ecotourism achieves balanced socio-cultural, economic, and environmental outcomes in TMCLs. Looking forward, a regenerative approach to ecotourism development could further ensure that these outcomes are not only sustained but enhanced. By striving for net positive contributions to the community and environment rather than growth in mere visitor numbers Vama Buzăului can avoid pitfalls of unbridled ecotourism expansion and remain on a development pathway that continuously improves its socio-ecological systems, securing a thriving future for the area.

## Data Availability

No datasets were generated or analysed during the current study.
